# Cross-sectional analysis of risk factors associated with *Mugil cephalus* in retail fish markets concerning methicillin-resistant *Staphylococcus aureus* and *Aeromonas hydrophila*

**DOI:** 10.3389/fcimb.2024.1348973

**Published:** 2024-02-02

**Authors:** Amira S. A. Attia, Rasha M. M. Abou Elez, Nashwa El-Gazzar, Shimaa S. Elnahriry, Ahmed Alfifi, Helal F. Al-Harthi, Dalal Hussien M. Alkhalifah, Wael N. Hozzein, Hassan Mohmoud Diab, Doaa Ibrahim

**Affiliations:** ^1^ Department of Veterinary Public Health, Faculty of Veterinary Medicine, Zagazig University, Zagazig, Egypt; ^2^ Department of Zoonoses, Faculty of Veterinary Medicine, Zagazig University, Zagazig, Egypt; ^3^ Department of Botany and Microbiology, Faculty of Science, Zagazig University, Zagazig, Egypt; ^4^ Department of Bacteriology, Mycology and Immunology, Faculty of Veterinary Medicine, University of Sadat City, Sadat City, Egypt; ^5^ Department of Public Health, College of Veterinary Medicine, King Faisal University, Al-Ahsa, Saudi Arabia; ^6^ Department of Biology, Turabah University College, Taif University, Taif, Saudi Arabia; ^7^ Department of Biology, College of Science, Princess Nourah bint Abdulrahman University, Riyadh, Saudi Arabia; ^8^ Botany and Microbiology Department, Faculty of Science, Beni-Suef University, Beni-Suef, Egypt; ^9^ Department of Animal and Poultry Health and Environment, Faculty of Veterinary Medicine, South Valley University, Qena, Egypt; ^10^ Department of Nutrition and Clinical Nutrition, Faculty of Veterinary Medicine, Zagazig University, Zagazig, Egypt

**Keywords:** mullet, *S. aureus*, multidrug resistant, virulence gene, MAR index, aerolysin gene, hemolysin gene

## Abstract

**Introduction:**

*Aeromonas hydrophila* and methicillin-resistant *Staphylococcus aureus* (MRSA) are potent bacterial pathogens posing major hazards to human health via consuming fish harboring these pathogens or by cross-contamination beyond the contaminated environment. The aim of this study was to determine risk variables associated with the presence of certain pathogenic bacteria from *Mugil cephalus* fish in retail markets in Egypt. The virulence genes of *A. hydrophila* and *S. aureus* were also studied. Furthermore, the antibiotic sensitivity and multidrug resistance of the microorganisms were evaluated.

**Methods:**

In a cross-sectional investigation, 370 samples were collected from mullet skin and muscle samples, washing water, fish handlers, knives, and chopping boards. Furthermore, fish handlers’ public health implications were assessed via their response to a descriptive questionnaire.

**Results:**

*S. aureus* and *Aeromonas* species dominated the investigated samples with percentages of 26.76% and 30.81%, respectively. Furthermore, *A. hydrophila* and MRSA were the predominant recovered bacterial pathogens among washing water and knives (53.85% and 46.66%, respectively). The virulence markers *aerA* and *hlyA* were found in 90.7% and 46.5% of *A. hydrophila* isolates, respectively. Moreover, the virulence genes *nuc* and *mec* were prevalent in 80% and 60% of *S. aureus* isolates, respectively. Antimicrobial susceptibility results revealed that all *A. hydrophila* isolates were resistant to amoxicillin and all MRSA isolates were resistant to amoxicillin and ampicillin. Remarkably, multiple drug resistance (MDR) patterns were detected in high proportions in *A. hydrophila* (88.37%) and MRSA (100%) isolates. The prevalence of *Aeromonas* spp. and *S. aureus* had a positive significant correlation with the frequency of handwashing and use of sanitizer in cleaning of instruments. MRSA showed the highest significant prevalence rate in the oldest age category.

**Conclusion:**

The pathogenic bacteria recovered in this study were virulent and had a significant correlation with risk factors associated with improper fish handling. Furthermore, a high frequency of MDR was detected in these pathogenic bacteria, posing a significant risk to food safety and public health.

## 1 Introduction

Fish is a main part of the human diet, offering good-quality animal protein besides its health benefits, comprising higher contents of omega-3, fatty acids, and vitamin D. Fish accounts for at least 20% of the protein ingested by one-third of the global population (Tørris et al., 2018; FAO, 2022; Alhawas et al., 2023; Kishawy et al., 2022). *Mugil cephalus* was ranked the second most important species following *Tilapia* species and considered the most commercially crucial fish in Egypt’s food supply due to its higher market acceptance and higher growth rates (Suloma and Ogata, 2006; El-Sayed, 2017; Alandiyjany et al., 2022; Morshdy et al., 2022). Egypt is eighth in the world in aquaculture production as it produces approximately 1.54% of total cultured fish all over the world and approximately 73.8% of total African production (FAO, 2012). Egypt is the world’s top producer of cultured *Mugil*, where *Mugil* and *Tilapia* aquacultures contribute 85.1% of total aquaculture production (El-Sayed, 2017; Abd El-Hamid et al., 2021; Ibrahim et al., 2021).

On the other hand, fish is considered a reservoir of pathogenic bacteria related to human illness resulting from consumption of contaminated raw or undercooked fish (Novotny et al., 2004). One of the bacterial pathogens affecting fish is *Aeromonas* species, which is ubiquitous, primarily causing outbreaks in fish farming. Additionally, these pathogens can enter fish markets via people or equipment and then survive and spread from contaminated fish workers’ hands to fish during handling and processing. Furthermore, water is also a vehicle for the transmission of these infectious agents. People can get *Aeromonas* infections by ingesting pathogenic organisms in contaminated food or water, resulting in a variety of extraintestinal infections ranging from gastroenteritis to septicemia (Igbinosa et al., 2012). However, cross-contamination and contact with contaminated water can cause microbial pollutants to spread into fresh fish or fishery products with circumstantial risks for consumers’ safety (Bedane et al., 2022). With special reference to *Aeromonas hydrophila*, which is a pathogen of public health significance, its pathogenicity has been linked to extracellular, cytotoxic, hemolytic, and enterotoxic enzymes such as aerolysin and hemolysin, whose genes are considered reliable markers in either human or fish (Soler et al., 2002; Umesha et al., 2011). Infection with *A. hydrophila* produces septicemia, ulcerative diseases, hemorrhagic diseases, lesions, and scale loss, leaving infected live fish useless and resulting in significant mortality rates in all fish species (Forhad et al., 2013). They transmit the zoonotic disease aeromoniasis to humans through eating of contaminated fish and water, causing serious diseases such as meningitis, septic arthritis, gastroenteritis with diarrhea, skin infections, and bacteremia (Stratev and Odeyemi, 2017; Abdel-Latif and Khafaga, 2020).

*Staphylococcus aureus* is widely found asymptomatically in the skin, mucous membranes, and noses of 25% of healthy individuals and various animal species. However, it is not considered a typical part of fish microflora; therefore, its presence on fish indicates either postharvest contamination or a disease occurrence in fish (Austin and Austin, 2007; Sivaraman et al., 2017). In addition, *S. aureus* is a prevalent foodborne pathogen regarded as a major human pathogen responsible for diseases such as staphylococcal food poisoning, toxic shock syndrome, bacteremia, and pneumonia (Crago et al., 2012; Chieffi et al., 2020). Methicillin-resistant *S. aureus* (MRSA) has been identified in aquatic animals, fish markets, and infected handlers (Solano et al., 2013; Abdel-Raheem et al., 2023). It can spread under unhygienic circumstances or through contaminated processing instruments or infected persons. People are asymptomatic carriers; thus, food handlers’ hygiene practices are partially responsible for increased cross-contamination and the ongoing propagation of MRSA (Hammad et al., 2012; Ferreira et al., 2014). However, MRSA was a causative agent for developing veterinary and zoonotic illness with cross infection and transmission from fish handlers to fish and vice versa (Sivaraman et al., 2022).

Several risk factors associated with the prevalence of these bacteria have been described, but environmental conditions, microbial quality of water, improper postharvest management, and processing through infected hands and surfaces during fish evisceration (Chintagari et al., 2017) are crucial features for pathogen transmission. Also, the handlers’ health conditions, working habits, personal hygiene such as frequency of washing hands, and gender and age and use of hand sanitizers are responsible for increased bacterial contamination and higher bacterial incidence rates (Ferreira et al., 2014).

Diseases caused by antibiotic-resistant foodborne pathogens such as *Aeromonas* species and MRSA are among the potential bacterial fish diseases of public health significance identified from fish (Novoslavskij et al., 2016; Agüeria et al., 2018). According to the World Health Organization (WHO), antimicrobial resistance (AMR) is one of the top 10 worldwide public health problems (World Health Organization, 2021). Moreover, there is a fundamental association between AMR and the usage of antibiotics in aquaculture as the latter are used to treat diseased fish and enhance growth and productivity (Landoni and Albarellos, 2015; Elfaky et al., 2022). Following that, long-term antibiotic usage and misuse caused antibiotics to enter the environment, resulting in the development of resistant bacteria and the formation of AMR in aquatic bacteria (Hossain and Heo, 2021; Aljazzar et al., 2022). Multiple drug resistance (MDR) is a serious problem with serious consequences for public health that can be generated in bacteria through several mechanisms, such as the accumulation of multiple genes coding for drug resistance within a cell, which typically occurs on resistance plasmids or may be due to an increase in genes coding for multidrug efflux pumps (Bendary et al., 2022; Catalano et al., 2022).

Given the widespread consumption of *M. cephalus* fish in Egypt (Suloma and Ogata, 2006; Morshdy et al., 2022), surveillance studies to determine the safety of ingested fish are required. As a result, the purpose of this study was to look into the risk variables that were linked to the prevalence of pathogenic bacteria isolated from different samples collected from retail marketplaces of fresh *M. cephalus* fish in Sharkia Governorate, Egypt. Furthermore, simplex PCR was performed to evaluate the expression of virulence genes in identified *A. hydrophila* and *S. aureus* isolates. The antimicrobial susceptibility of isolated bacteria and MDR patterns were also investigated.

## 2 Material and methods

### 2.1 Sample collection and study design

For this study, 370 samples were chosen from various fish retail marketplaces in Sharkia Governorate, Egypt. Apparently healthy *M. cephalus* fish with no clinical signs of infections were selected. Mullet fish skin and dorsal muscle samples (120 each) were collected. Swabs were randomly taken from the hands of seafood sellers (29) and processors (11). Additionally, swab samples of knives and chopping boards (30 each) were obtained from the same markets where the fish was purchased. Furthermore, 30 fish washing water samples were collected during the period from May 2022 to March 2023.

Swabbing the surface of the fish, the fish handler’s hands, the blades, and the chopping boards with a sterile swab immersed in pre-enrichment buffer peptone water broth (BPW) was the sample procedure. A total of 100 mL of fish washing water was also collected in sterile screw-capped colorless glass vials. Mullet fish were placed in sterilized polyethylene bags. All samples were labeled and transferred aseptically in an icebox immediately with no delay to the laboratory of the Veterinary Public Health Department, Faculty of Veterinary Medicine, Zagazig University, in accordance with ISO 7218:2007 requirements (ISO, 2013). The dorsal muscle of surface-sterilized fish samples was obtained in the laboratory under completely aseptic conditions (APHA, 2001). Prior to sampling, fish handlers completed a questionnaire about their age, gender, job title, working hours per day, time spent in fish markets, sanitary practices such as frequency of handwashing, use of hand sanitizer, cleaning and disinfecting of utensils, history of skin infection, and use of non-prescription antibiotics. The data were collected utilizing 40 questionnaire forms.

### 2.2 Isolation and identification for *Aeromonas* spp. and *S. aureus*

Twenty-five (g) of fish muscle and 25 mL of fish washing water samples were added into 225 mL of BPW (Oxoid, CM509) and incubated at 37°C for 6 h. To isolate *Aeromonas* spp., 1 mL of each pre-enriched broth was transferred to 10 mL of enrichment tryptic soy broth and incubated for 24 h at 37°C (Cruichshank et al., 1975) and then streaked on *Aeromonas* agar base media (LAB, 167) and incubated at 37°C for 24 h. The suspected *Aeromonas* isolates were subcultured on nutrient agar for further identification using morphological characters and biochemically by loading 100 μL of the microbial suspension onto API 20 E kits (bioMérieux, France) per the manufacturer’s instructions (Ashiru et al., 2011).

As previously described, Baird-Parker agar mixed with an egg yolk tellurite emulsion (Difco Laboratories, Detroit, MI) was used to isolate *S. aureus* (APHA, 2001). Presumptive colonies were subcultured on blood agar plates (Difco Laboratories, Detroit, MI) and incubated at 37°C for 24 h after inoculation. *S. aureus* colonies were recognized morphologically by their Gram-positive grapelike clusters and biochemically based on their positive reactions in catalase and coagulase tests (Quinn et al., 2011). *S. aureus* isolates were subcultured onto oxacillin resistance screening agar base (ORSAB) media for 24 h at 37°C for morphological detection and differentiation of MRSA by their deep blue coloration (Mohammed et al., 2020).

### 2.3 Molecular characterization of *A. hydrophila* and *S. aureus* isolates

For confirming the phenotypic identity of *A. hydrophila* isolates, they were tested for aerolysin (*aer*) and hemolysin (*hly*) genes using PCR. Furthermore, the phenotypically detected *S. aureus* isolates were subjected to a simplex PCR assay for detection of *Staphylococcus* genus–specific (*23S rRNA*) and *S. aureus* species–specific (*nuc* gene) markers. Methicillin resistance was also determined by detecting the *mecA* gene at the Biotechnology Unit of the Poultry Animal Health Research Institute’s National Laboratory for Veterinary Quality Control in Doki, Giza, Egypt. QIAamp^®^ DNA Mini Kit (Cat. No. 51304, Qiagen) was used to extract and prepare genomic DNA using the proteinase K technique. According to Ahmed et al. (2018), the PCR amplification was performed on extracted DNA using primers detailed in **Table 1**.

**Table 1:** Primer sequences, target genes, amplicon sizes, and cycling conditions used in the study.

Target gene	Primer sequences 5′ ^ـــــــــــــــــــــــــــــــــــــــــــــــــــــ^ 3′	Amplified segment (bp)	Primary denaturation	Amplification (35 cycles)			Final extension	Reference
				Secondary denaturation	Annealing	Extension		
***aer A***	ACAGCCAATATGTCGGTGAAG GTCACCTTCTCGCTCAGGC	326	95°C 5 min	94°C 1 min	52°C 1 min	72°C 1 min	72°C 10 min	Singh et al. (2008)
***hly A***	CTATGAAAAAACTAAAAATAACTG CAGTATAAGTGGGGAAATGGAAAG	1,500	95°C 5 min	94°C 1 min	55°C 1 min	72°C 3 min	72°C 10 min	Yousr et al. (2007)
***23S rRNA***	AC GGAGTTACAAAGGACGAC AGCTCAGCCTTAACGAGTAC	1,250	94°C 5 min	94°C 30 s	55°C 40 s	72°C 12 min	72°C 12 min	Bhati et al., 2016
***nuc***	ATATGTATGGCAATCGTTTCAAT GTAAATGCACTTGCTTCAGGAC	395	94°C 30 s	94°C 30 s	55°C 40 s	72°C 40 s	72°C 7 min	Gao et al., 2011
***mecA***	GTA GAA ATG ACT GAA CGT CCG ATAA CCA ATT CCA CAT TGT TTC GGT CTA A	310	94°C 5 min	94°C 30 s	50°C 30 s	72°C 30 s	72°C 7 min	McClure et al., 2006

### 2.4 Antibiotic susceptibility test

*A. hydrophila*, *S. aureus*, and MRSA isolates were evaluated for antibiotic resistance using the disk diffusion method (Oxoid, UK) in accordance with the recommendations of Clinical and Laboratory Standard Institute guidelines (CLSI, 2013). The 10 antibiotics tested (bioMérieux F6980, Marcy-l’Étoile, France) were ampicillin (AM, 10 µg), chloramphenicol (C, 30 µg), ciprofloxacin (CIP, 5 µg), enrofloxacin (ENR, 5 µg), erythromycin (ERY, 15 µg), nalidixic acid (NA, 30 µg), gentamicin (GN, 10 µg), amoxicillin (AXE, 25 µg), tetracycline (TE, 30 µg), and trimethoprim/sulfamethoxazole (SXT, 25 µg). Bacterial isolates were classified as resistant (R), intermediate (I), or susceptible (S) by measurement of inhibition zone diameters as prior criteria of CLSI guidelines. Multidrug-resistant isolates demonstrated resistance to at least one antimicrobial drug in three or more different antimicrobial classes (Magiorakos et al., 2012). Furthermore, the multiple antibiotic resistance (MAR) index for isolates was determined using the formula a/b, where “a” represents the number of antibiotics to which an isolate was resistant and “b” represents the total number of drugs tested (Krumperman, 1983).

### 2.5 Statistical analysis

The IBM SPSS software (SPSS statistical system package) was used to analyze the data in this study. To describe the relationship between variables, a basic descriptive analysis was utilized. The chi-square test was used to identify significant correlations (P < 0.05) (McHugh, 2013).

## 3 Results

### 3.1 Prevalence of bacterial pathogens isolated from various sources in fish markets

A total of 370 samples of fish skin, fish muscle, fish washing water, knives, chopping boards, and fish handlers were analyzed for the presence of *Aeromonas* spp., *A. hydrophila*, *S. aureus*, and MRSA (**Table 2**). *Aeromonas* species were detected in 114 (30.81%) of the samples. Furthermore, *Aeromonas* spp. were most frequent in washing water (43.33%), followed by fish skin (41.6%), knives (36.66%), chopping boards (33.33%), fish handlers (25%), and fish muscle (16.66%). Biochemical testing detected 43 (37.72%) *A. hydrophila* isolates from 114 *Aeromonas* spp. isolates. Similarly, *A. hydrophila* was most frequent in washing water (53.85%), followed by fish handlers and skin (40% each). According to **Table 2**, *S. aureus* was found in 99 of 370 investigated samples (26.76%) and was most commonly found in knives (14, 46.66%), fish handlers (15, 37.5%), chopping boards (10, 33.33%), fish skin (38, 31.66%), and muscle (7, 5.83%). In regard to MRSA, it was found that 15.15% of *S. aureus* isolates (15 out of 99) showed deep blue color on ORSAB media, which is the phenotypic appearance of MRSA. However, MRSA was most frequent in fish washing water, chopping boards, and fish handlers (20% each). In terms of sample sources, **Table 2** revealed statistically significant changes in *Aeromonas* spp. and *S. aureus* prevalence with different sources, but not in the case of *A. hydrophila* and MRSA.

**Table 2:** Prevalence of bacterial pathogens isolated from different sources collected from fish markets in Egypt.

Sampling units	No. of samples	Positive for bacterial pathogens							
		*Aeromonas* sp.		*A. hydrophila*		*S. aureus*		*Staph MRSA*	
		No.^1^	%	No.^2^	%	No.^1^	%	No.^2^	%
1. ***M cephalus*** a. Skin b. Muscle	120 120	50 20	41.6 16.66	20 5	40 25	38 7	31.66 5.83	4 1	10.53 14.3
2. Washing water	30	13	43.33	7	53.85	15	50	3	20
3. Knives 4. Chopping boards 5. Fish handler’s	30	11	36.66	4	36.36	14	46.66	2	14.28
	30	10	33.33	3	30	10	33.33	2	20
	40	10	25	4	40	15	37.5	3	20
TOTAL	370	114	30.81	43	37.72	99	26.76	15	15.15
Chi-square value		21.30*		3.21		45.63*		1.37	

^1%^ calculated according to the number of examined samples.

^2%^ calculated according to the number of positive samples.

* Significant level of chi-square value was considered at P < 0.05.

### 3.2 Molecular characterization of *A. hydrophila* and *S. aureus* isolates

**Figure 1:**
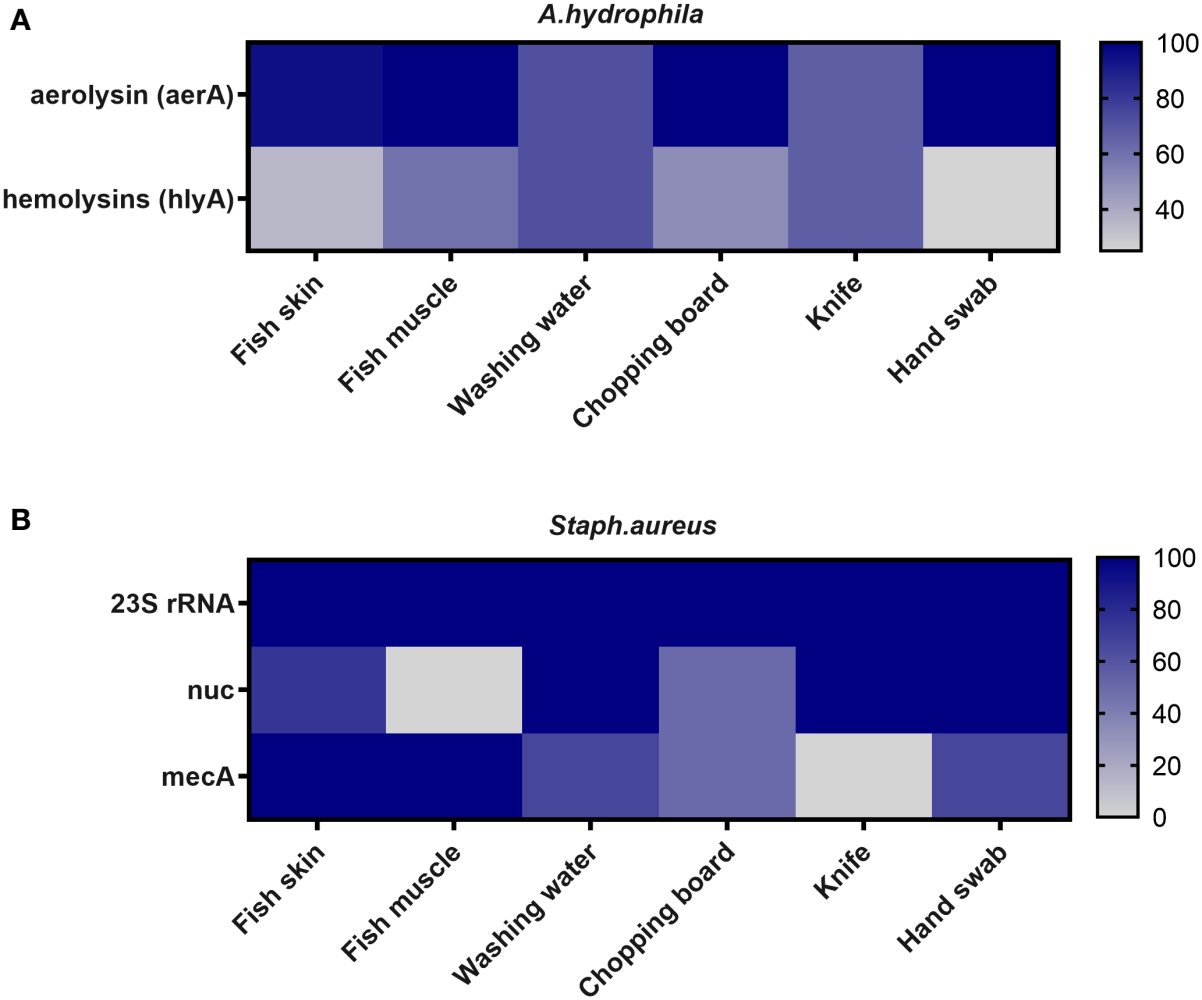
Frequency of virulence genes of **(A)** *A. hydrophila* and **(B)** *S. aureus* recovered from different sources in retail fish markets.

**Figure 1** illustrates the molecular identification of selected virulence genes in *A. hydrophila* and *S. aureus* isolates. The aerolysin (*aerA*) gene was found in 90.7% of the examined *A. hydrophila* (39 out of 43), with the highest prevalence in fish muscles, knives, and hand swabs (100%). Furthermore, the hemolysin (*hlyA*) gene was found in 46.5% of the same tested isolates, with the highest percentages (71.4% and 66.67%) found in washing water and chopping boards, respectively (**Figure 1A**; **Supplementary Figure 1**).

The presence of virulence genes (*23S rRNA*, *nuc*, and *mec*) in *S. aureus* isolates was checked (**Figure 1B**; **Supplementary Figure 2**). The *23S rRNA* gene was found in all *S. aureus* strains (100%). The *nuc* and *mec* genes were found in 80% (12/15) and 60% (9/15) of *S. aureus* strains, respectively. The *nuc* gene was found in 100% of the fish washing water, chopping boards, and hand swab isolates. Notably, the *mec* gene was present in 100% of the fish skin and muscle.

### 3.3 The impact of risk factors on the prevalence of isolated bacterial pathogens

**Table 3** shows the prevalence of *Aeromonas* spp., *A. hydrophila*, *S. aureus*, and MRSA positivity based on various variables. In terms of gender, the questionnaire survey included 40 respondents (25 men and 15 women). Men had a higher prevalence of *Aeromonas* spp., *A. hydrophila*, and MRSA than women (32% versus 13%, 16% compared to 0%, and 8% versus 6.6%, respectively), with no significant differences. According to the results of the questionnaire, 8 (20%) of the respondents were under the age of 20, 17 (42.5%) were between the ages of 21 and 30, and the remaining 15 (37.5%) were above the age of 31. When compared to the other categories, the age category “>30 years” had the highest prevalence rate of *Aeromonas* spp., *A. hydrophila*, *S. aureus*, and MRSA (33.3%, 26.6%, 46.6%, and 20%, respectively), with significant associations between *A. hydrophila* and MRSA prevalence in age category “<20 years” (0% versus 26.6% and 0% versus 20%; p < 0.05). In terms of job title and working hours per day, fish processors were represented by 27.5% of respondents, and 60% of respondents spent 6–10 h per day in fish markets. These groups had higher no significant positivity rates for *Aeromonas* spp., *S. aureus*, and MRSA than those selling fish (27.2% compared to 24.1%; 45.4% versus 34.5%; and 9.1% versus 6.9%). *Aeromonas* spp. and *A. hydrophila* positivity was higher in the non–washing hands group (23 respondents) than in the washing hands group (39.1% versus 5.9% and 17.4% versus 0%; p< 0.05). Furthermore, a stronger significant correlation was identified between the prevalence of *Aeromonas* spp. and *S. aureus* and “frequency of washing hands” in the once group (19 respondents) than in the other two groups (36.8% versus 20% versus 0% and 52.6% versus 33.3% versus 0%; p < 0.05). In terms of hand sanitizer use, 35% of those who used sanitizer had considerably lower *S. aureus* prevalence than the remaining 65% who did not (0% versus 57.6%; p < 0.05).

**Table 3:** Risk factors associated with the frequency of bacterial pathogens found in Egyptian fish markets.

Risk factor	Class			Positive for bacterial pathogens (%)			
		N	*%*	*Aeromonas* spp.	*A. hydrophila*	*S. aureus*	*Staph. MRSA*
Gender							
	Male	25	62.5	32.0	16.0	36.0	8.0
	Female	15	37.5	13.3	0.0	40.0	6.6
Age (years)							
	≤20	8	20	12.5	0.0*	12.5	0.0*
	21–30	17	42.5	23.5	0.0	35.3	0.0
	>30	15	37.5	33.3	26.6	46.6	20.0
Job title							
	Fish seller	29	75.5	24.1	10.3	34.5	6.9
	Fish processor	11	27.5	27.2	9.1	45.4	9.1
Working hours/day							
	<5 h	16	40	12.5	6.3	43.7	6.3
	6–10 h	24	60	33.3	12.5	33.3	8.3
Washing hands before and/or after fish handling							
	Yes	17	42.5	5.9*	0.0*	23.5	5.9
	No	23	57.5	39.1	17.4	47.8	8.7
Frequency of washing hands							
	Once	19	47.5	36.8*	15.8	52.6*	10.5
	Twice	15	37.5	20.0	6.6	33.3	6.6
	≥Thrice	6	15	0.0	0.0	0.0	0.0
Using hand sanitizers							
	Yes	14	35	14.3	0.0	0.0*	0.0
	No	26	65	30.8	15.4	57.6	11.5
History of skin infection							
	Yes	26	65	30.8	15.4	50.0*	11.5
	No	14	35	14.3	0.0	14.3	0.0
Using unprescribed antibiotics							
	Yes	17	65.38	41.2	17.6	52.9	17.6
	No	9	34.62	11.1	11.1	33.3	0.0
Using sanitizer in cleaning of instruments							
	Yes	18	45	5.6*	0.0*	16.7*	5.6
	No	22	55	40.9	18.2	54.5	9.1

*Significant level of chi-square value was considered at P < 0.05.

The prevalence rates of all isolated bacteria were higher in the group with a history of skin infection (26 respondents) with a significant difference in *S. aureus* positivity than the other group (50% versus 14.3%; p < 0.05). Among them, the group using unprescribed antibiotics (17 respondents) also showed a higher prevalence of all bacterial isolates with no statistical significance difference than the “not using antibiotics” group. In the assessed fish markets, 45% of respondents who used sanitizer to clean their instruments had considerably lower prevalence of *Aeromonas* spp., *A. hydrophila*, and *S. aureus* than the non-sanitizer group (5.6% versus 40.9%; 0% compared to 18.2%; p < 0.05).

### 3.4 Profile of antibiotic susceptibility of isolated bacterial pathogens

**Figure 2:**
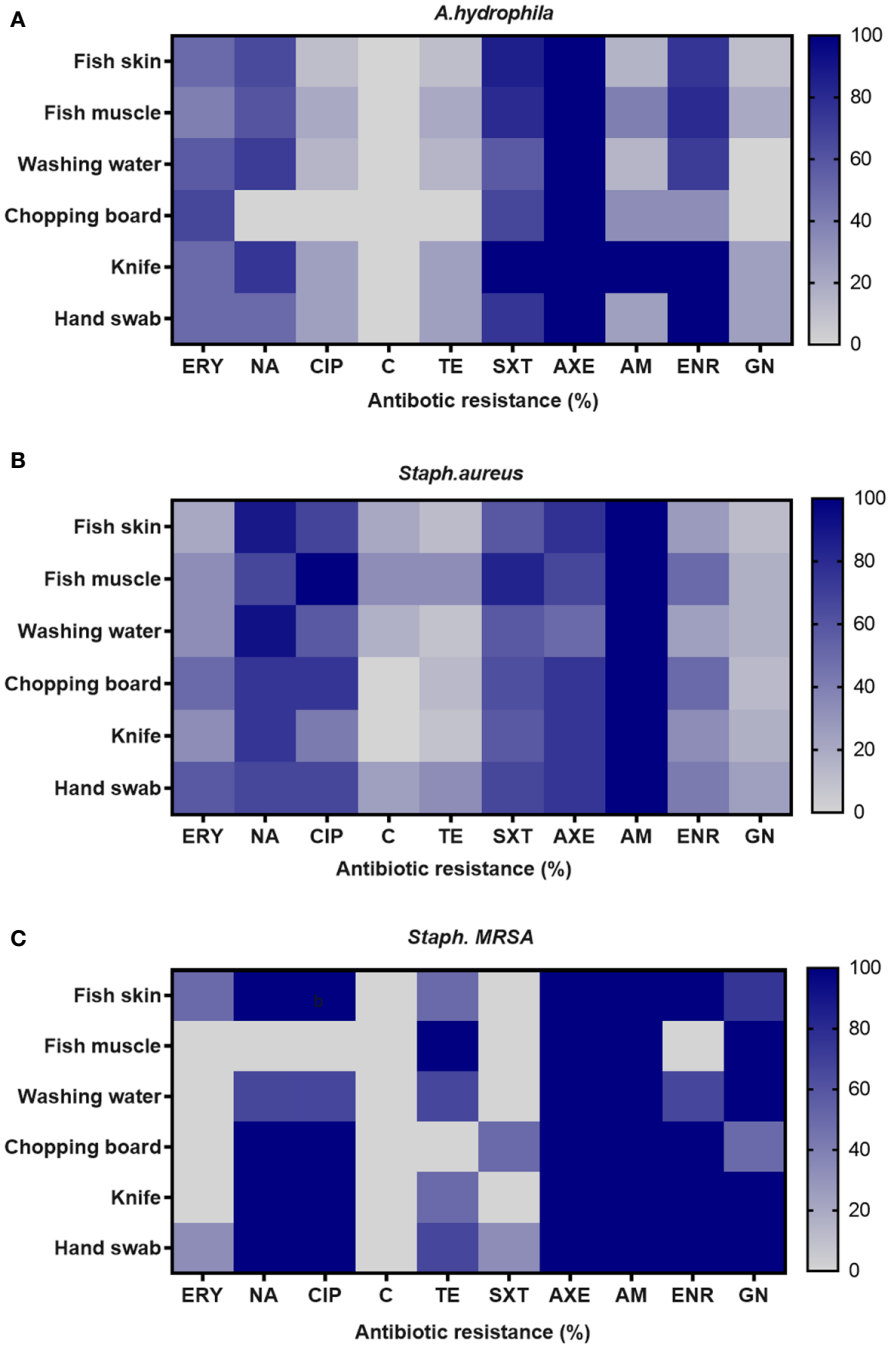
Frequency of antimicrobial resistance of **(A)** *A. hydrophila*, **(B)** *S. aureus*, and **(C)** *Staph. MRSA* in different sources in examined fish markets.

**Table 4** shows the distribution of antibiotic resistance patterns of *A. hydrophila*, *S. aureus*, and MRSA against 10 antimicrobial drugs. All *A. hydrophila* isolates tested positive for AXE (100%) and TE (88.37%). The isolates were more sensitive to C (90.69%) and GN (88.37%). The resistance profiles of the 84 *S*. *aureus* isolates revealed varying levels of resistance against the tested antibiotics, with AM (100%) showing the highest resistance, followed by NA and AXE (80.95% and 71.43%, respectively), but those same strains exhibited high sensitivity to GN (84.52%) and TE (70.24%). In terms of MRSA resistance, the antibiotics with the highest rates of resistance were AXE and AM (100%). Also, MRSA showed high resistance (86.66% each) to the quinolones group with significant correlation at P < 0.05 in different sources (**Supplementary Table 1** and **Figure 2**). MRSA has a high sensitivity to C (100%) and SXT (93.33%).

**Table 4:** *A. hydrophila*, *S. aureus*, and *MRSA* antibiotic resistance patterns found in fish markets.

Antimicrobial class	Antibiotics	Antibiotic resistance patterns								
		*A. hydrophila* N (%)			*S. aureus* N (%)			*Staph. MRSA* N (%)		
		*(n = 43)*			*(n = 84)*			*(n = 15)*		
		S	I	R	S	I	R	S	I	R
**Macrolides**	**Erythromycin**	0(0)	21(48.8)	22(51.16)	0(0)	56(66.67)	28(33.33)	11(73.3)	1(6.66)	3(20)
**Quinolones**	**Nalidixic acid**	8(18.60)	9(20.9)	26(60.5)	0(0)	16(19.05)	68(80.95)	1(6.66)	1(6.66)	13(86.66)
	**Ciprofloxacin**	14(32.55)	23(53.48)	6(13.95)	29(34.52)	0(0)	55(65.48)	1(6.66)	1(6.66)	13(86.66)
	**Enrofloxacin**	5(11.63)	5(11.63)	33(76.74)	42(50)	14(16.67)	28(33.33)	3(20)	0(0)	12(80)
**Phenols**	**Chloramphenicol**	39(90.69)	4(9.31)	0(0)	47(55.95)	23(27.38)	14(16.67)	15(100)	0(0)	0(0)
**Tetracycline**	**Tetracycline**	5(11.63)	0(0)	38(88.37)	59(70.24)	12(14.28)	13(15.48)	3(20)	4(26.66)	8(52.94)
**Sulfonamides**	**Trimethoprim–sulfamethoxazole**	9(20.93)	0(0)	34(79.07)	32(38.09)	0(0)	52(61.91)	14(93.33)	0(0)	1(6.66)
**Cephalosporine**	**Amoxicillin**	0(0)	0(0)	43(100)	24(28.57)	0(0)	60(71.43)	0(0)	0(0)	15(100)
**Penicillins**	**Ampicillin**	7(16.3)	27(62.8)	9(20.9)	0(0)	0(0)	84(100)	0(0)	0(0)	15(100)
**Aminoglycosides**	**Gentamicin**	38(88.37)	0(0)	5(11.63)	71(84.52)	0(0)	13(15.48)	2(13.33)	0(0)	13(86.66)

S, sensitive; I, intermediate sensitive; R, resistant

**Table 5**; **Figure 3A** demonstrate the MDR and MAR indexes of isolated bacterial species. Multidrug resistance was found in 88.37% of *A. hydrophila* isolates, with a MAR index ranging from 0.2 to 0.8, with a mean of 0.5. Knives had the highest MAR index (**Figure 3B**), followed by hand swabs, fish muscle, and skin. All *S. aureus* and MRSA strains were found to be multidrug resistant, with MAR indexes of 0.53 and 0.45, respectively. An *S. aureus* MAR index of 0.6 was found in fish muscle and hand swabs (**Figure 3C**), while an MRSA index of 0.53 was found in fish washing water (**Figure 3D**).

**Table 5:** Distribution of different patterns of antibiotic resistance in isolates of *A. hydrophila*, *S. aureus*, and *Staph. MRS*.

Pathogen	Number of antimicrobial classes	Number of antibiotics	Resistance patterns^1^	MDR (%)^2^	MAR index^3^		
A. hydrophila (n = 43)							
	3	3	TE, AXE, ENR	2 (4.6)	0.3	
	5	6	ERY, NA, TE, SXT, AXE, ENR	12 (27.9)	0.6	
	4	4	TE, SXT, AXE, ENR	2 (4.6)	0.4	
	7	9	ERY, NA, CIP, TE, SXT, AXE, AM, ENR, GN	1(2.3)	0.9	
	6	8	ERY, NA, CIP, TE, SXT, AXE, AM, ENR	3 (6.9)	0.8	
	3	3	TE, SXT, AXE	2 (4.6)	0.3	
	4	4	ERY, TE, AXE, ENR	1(2.3)	0.4	
	5	6	NA, TE, SXT, AXE, ENR, GN	2 (4.6)	0.6	
	4	5	NA, TE, SXT, AXE, ENR	2 (4.6)	0.5	
	5	5	NA, TE, SXT, AXE, AM	2 (4.6)	0.5	
	2	2	SXT, AXE	–	0.2	
	1	1	AXE	–	0.1	
	6	7	ERY, CIP, TE, SXT, AXE, AM, ENR	1(2.3)	0.7	
	4	4	NA, TE, AXE, AM	1(2.3)	0.4	
	5	5	TE, SXT, AXE, ENR, GN	1(2.3)	0.5	
	5	5	ERY, TE, SXT, AXE, ENR	1(2.3)	0.5	
	4	4	ERY, TE, AXE, AM	1(2.3)	0.4	
	2	2	SXT, AXE	–	0.2	
	5	7	NA, CIP, TE, SXT, AXE, AM, ENR	1(2.3)	0.7	
	6	7	ERY, NA, TE, SXT, AXE, ENR, GN	1(2.3)	0.7	
	5	5	ERY, NA, TE, SXT, AXE	1(2.3)	0.5	
	2	3	NA, AXE, ENR	1(2.3)	0.3	
	2	2	AXE, ENR	–	0.2	
	2	2	TE, AXE	–	0.2	
					38 (88.37)		
S. aureus (n = 84)							
	6	7	ERY, NA, CIP, C, SXT, AXE, AM	5 (5.9)	0.7	
	4	4	NA, SXT, AXE, AM	7 (8.3)	0.4	
	3	3	NA, AXE, AM	8 (9.5)	0.3	
	4	4	ERY, NA, AXE, AM	2 (2.4)	0.4	
	4	5	NA, CIP, SXT, AXE, AM	12(14.3)	0.5	
	4	5	NA, SXT, AXE, AM, ENR	3 (3.6)	0.5	
	3	4	NA, CIP, AXE, AM	5 (5.9)	0.4	
	5	7	NA, CIP, C, SXT, AXE, AM, ENR	2 (2.4)	0.7	
	4	5	CIP, TE, AXE, AM, ENR	3(3.6)	0.5	
	5	5	CIP, TE, AXE, AM, GN	1(1.2)	0.5	
	4	6	NA, CIP, SXT, AXE, AM, ENR	5 (5.9)	0.6	
	5	6	ERY, NA, SXT, AXE, AM, ENR	2 (2.4)	0.6	
	5	6	NA, CIP, C, SXT, AXE, AM	2 (2.4)	0.6	
	3	3	AXE, AM, GN	2 (2.4)	0.3	
	5	6	ERY, NA, CIP, SXT, AXE, AM	5 (5.9)	0.6	
	5	6	NA, CIP, C, AXE, AM, GN	1(1.2)	0.6	
	5	7	NA, CIP, C, TE, AM, ENR, GN	1(1.2)	0.7	
	7	8	ERY, CIP, TE, SXT, AXE, AM, ENR, GN	2 (2.4)	0.8	
	7	9	ERY, NA, CIP, C, TE, SXT, AXE, AM, ENR	1(1.2)	0.9	
	4	5	ERY, TE, AXE, AM, ENR	1(1.2)	0.5	
	5	7	ERY, NA, CIP, SXT, AXE, AM, ENR	4 (4.8)	0.7	
	4	4	ERY, AXE, AM, GN	2 (2.4)	0.4	
	4	4	SXT, AXE, AM, ENR	2 (2.4)	0.4	
	6	7	NA, CIP, C, TE, AXE, AM, GN	1(1.2)	0.7	
	3	5	ERY, NA, AXE, AM, ENR	1(1.2)	0.5	
	6	7	ERY, CIP, TE, SXT, AXE, AM, ENR	1(1.2)	0.7	
	5	5	CIP, SXT, AXE, AM, GN	1(1.2)	0.5	
	6	7	ERY, CIP, TE, AXE, AM, ENR, GN	1(1.2)	0.7	
	7	8	ERY, NA, CIP, C, TE, AXE, AM, GN	1(1.2)	0.8	
					84(100)		
Staph. MRSA (n = 15)							
	6	6	ERY, CIP, TE, AXE, AM, GN	1 (6.66)	0.6	
	3	3	CIP, AXE, AM	2(13.33)	0.3	
	5	5	CIP, TE, AXE, AM, GN	1 (6.66)	0.5	
	5	5	ERY, CIP, AXE, AM, GN	2(13.33)	0.5	
	4	4	TE, AXE, AM, GN	1 (6.66)	0.4	
	6	6	CIP, TE, SXT, AXE, AM, GN	1 (6.66)	0.6	
	5	5	CIP, TE, AXE, AM, GN	2(13.33)	0.5	
	4	4	CIP, AXE, AM, GN	2(13.33)	0.4	
	5	5	CIP, TE, AXE, AM, GN	2(13.33)	0.5	
	3	3	AXE, AM, GN	1 (6.66)	0.3	
				15 (100)		

1 Ampicillin, AM; chloramphenicol, C; ciprofloxacin, CIP; enrofloxacin, ENR; erythromycin, ERY; nalidixic acid, NA; gentamicin, GN; amoxicillin, AXE; tetracycline, TE; trimethoprim/sulfamethoxazole, SXT.

2 MDR, multiple drug resistance.

3 MAR, multiple antibiotic resistance index.

**Figure 3:**
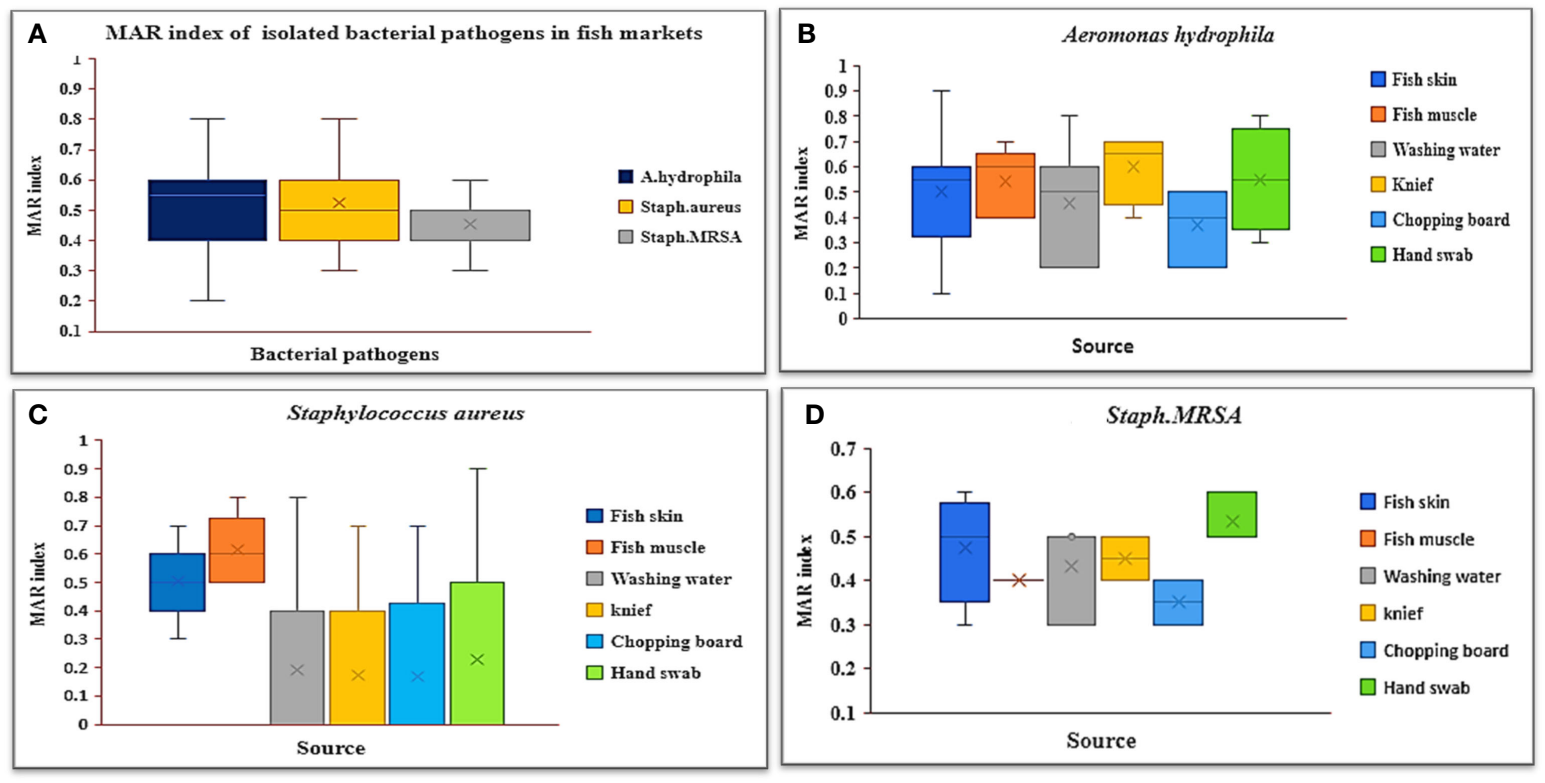
Box plots of the **(A)** MAR index of isolated bacterial pathogens recovered from different sources in retail fish markets, **(B)** MAR index of *A. hydrophila*, **(C)** MAR index of *S. aureus*, and **(D)** MAR index of *Staph. MRSA.*.

## 4 Discussion

*Aeromonas* species prevalence (30.81%) in the current study was lower than those previously reported for mullet fish in Egypt: 50% (Kishk et al., 2020), 62.5% (Abd El-Tawab et al., 2021), and 38% (Morshdy et al., 2022). In comparison, our discovery was higher than the reported 3.2% for *Aeromonas* spp. in Egyptian mullet fish by El-Hossary et al. (2023). Most *Aeromonas* species were found in washing water, followed by fish skin, knives, chopping boards, fish handlers, and fish muscle. These results were nearly equivalent to those obtained by Eid et al. (2022) in mullet fish skin (44%) and water samples (36%), both in Port Said, Egypt. Furthermore, El-Hossary et al. (2023) found a higher prevalence of *Aeromonas* spp. in hands (23.5%) than in fish muscle (11.9%). *Aeromonas* species were able to stick to hands and blades and could have come from fish or wash water, making them potential sources of cross-contamination (Thayumanavan et al., 2007). *A. hydrophila* is a zoonotic pathogen that can cause human sickness as well as a fish pathogen (Stratev and Odeyemi, 2016). The prevalence of *A. hydrophila* in fish (37.72%) was higher than that in mullet fish (1.2%; El-Hossary et al., 2023) but lower than the 54.2% reported by Abd El-Tawab et al. (2021). The highest level of *A. hydrophila* was identified in washing water, followed by fish handlers, fish skin, knives, chopping boards, and fish muscle. Prior research found a higher proportion in wash water than in hand swabs and chopping boards in fish outlets in India (Thayumanavan et al., 2007). In this study, however, the *A. hydrophila* isolation rate from fish muscle (25%) was similar to the previously assessed rate in El-Sharkia (28.6%; Attia et al., 2018) and El-Behera (25% Sonkol et al., 2020) but higher than the 20% reported by Kishk et al. (2020) and lower than rates reported in Dakahliya (37%; Ramadan et al., 2018) and Kafr El-sheik (30%; Sonkol et al., 2020). Spraying water on the fish and knives before or after cutting the fish, washing the knives and hands in the same water, and inappropriate container cleaning could all contribute to the higher prevalence of *A. hydrophila* in washing water. In all of the retail fish markets studied, handlers did not sanitize their hands or the boards after each usage. Furthermore, wooden chopping boards and knives remained a potential source of cross-contamination because sharp blades frequently left scars on the boards while processing fish, resulting in the adhesion of both minute muscle particles of the fish and organisms to the board (Thayumanavan et al., 2007).

The aerolysin (*aerA*) gene was found in 90.7% of *A. hydrophila* isolates studied. Our finding have been validated by previous studies that found a greater prevalence of the *aerA* gene (100%) in *A. hydrophila* isolated from fish in Egypt (Algammal et al., 2020; Elkamouny et al., 2020; Sonkol et al., 2020; Abd El-Tawab et al., 2021) but higher than the 70%, 80%, and 51.7% found in fish samples (Ramadan et al., 2018; Kishk et al., 2020; Mokhtar et al., 2023, respectively). While, hemolysin (hlyA) gene was discovered in 46.5% of A. hydrophila isolates, which was nearly similar to the previous studies in Egypt, (42%; Ramadan et al., 2018 and 44.4%; Sherif and Kassab, 2023), whereas higher than the 30% previously reported in Egypt (Sonkol et al., 2020) and lower than the prevalence rate of 100% (Algammal et al., 2020; Elkamouny et al., 2020; Abd El-Tawab et al., 2021) and 63.6% (Attia et al., 2018) reported in Egypt. These variances could be caused by the different species, geographical region, and selection era. Thus, the abundance of *A. hydrophila* bearing virulence genes discovered in our study suggests that these pathogenic bacteria constitute a major risk to public health (Abd-Elall et al., 2014).

In our investigation, *S. aureus* was found in 26.76% of all examined samples. These findings were exceptional, as evidenced by the presence of 2%–60% *S. aureus* in Iranian fish (Vaiyapuri et al., 2019) and 24.47% in Indian fish (Bujjamma and Padmavathi, 2015). The results of this study were more conclusive than the values of 4.80% in Ethiopia (Mitiku et al., 2022) and 16% in Iran (Arfatahery et al., 2016) but were less significant than those found in Egypt (31.8%; Mohammed et al., 2015), in Kerala (36.5%; Murugadas et al., 2016), and in Maiduguri (31.14%; Mohammed et al., 2020). The highest incidence of *S. aureus* isolates were found in knives (46.66%) and fish handlers (37.5%), according to our findings. These findings were supported by the other authors, who observed the highest frequency of *S. aureus* among fish handlers compared to other samples [33.3% (Grema et al., 2015) and 26.3% (Lim et al., 2023)] in the Sultanate of Oman. In Greek fish markets, Abrahima et al. (2010) discovered the highest frequency of *S. aureus* in knives (33%), followed by fish skin and muscle (24% each) and chopping boards (14%). In general, our findings were lower than those for fish (90%) and hands and surface swabs (75% each) but similar to that for water in India (50%) (Anjusha et al., 2022) and for fish in India (18.52%) (Sivaraman et al., 2022). In terms of hygiene, *S. aureus* is a pathogen of public health concern to consumers because of the presence of heat-stable enterotoxins that can cause foodborne poisoning, particularly when consuming raw or uncooked food (Da Silva et al., 2020).

MRSA is a zoonotic multidrug-resistant bacteria that increased morbidity and mortality in humans and animals (Dahms et al., 2014). Approximately 15% of *S. aureus* isolates tested positive for MRSA. Costa et al. (2015) discovered that MRSA was prevalent in 15% of processed fish. Our findings indicated a range of 0.95% to 13.4% MRSA in fish and its products in India (Vaiyapuri et al., 2019; Sivaraman et al., 2022). In this investigation, MRSA was discovered in 20% of the fish washing water, chopping boards, and fish handlers; 14.3% of muscle and knives; and 10.53% of fish skin samples. These rates were higher than previously reported rates of 15% in fish handlers, 4.5% in utensils, and 2.6% in fish skin (Grema et al., 2015); 5% in raw fish in India (Anjusha et al., 2022); and 2.7% in fish and 0% water samples in India (Sivaraman et al., 2022). Furthermore, our data from fish and environmental samples were lower than 35.2% to 23.5% in India (Murugadas et al., 2017), 100% in Nigeria (Arfatahery et al., 2016), and 50.78% in Iran (Mohammed et al., 2020). According to Anjusha et al. (2022), sewage contamination or unsanitary circumstances during fish handling and marketing may be the cause of the relatively highest prevalence of *S. aureus* and MRSA in our study’s fish washing water, fish handlers, and instruments. Additionally, according to Murugadas et al. (2017), water was most likely the cause of MRSA contamination in retail fish markets. Generally, the obtained results give evidence of the inadequate hygienic level of food safety and poor handling practices at fish markets. *Aeromonas* species, *A. hydrophila*, *S. aureus*, and MRSA prevalence differences between our studies and previous studies can be attributed to differences in sampling time, sample size, geographical location, fish species, post-capture contamination, and sanitary conditions during transportation, storage, and processing and differences in management and hygienic practices (Hafez et al., 2018).

The PCR study of *mecA* genes (encoding methicillin resistance) and *nuc* genes (encoding staphylococcal thermostable nuclease) is considered a quick MRSA strain identification approach (Sahebnasagh et al., 2014). The *23S rRNA* gene was found in 100% of the *S. aureus* isolates in this study, which was similar to recent findings from Turkey (Aksakal et al., 2022) and India (Murugadas et al., 2016). The *nuc* gene was detected in 80% of screened *S. aureus* isolates. The highest prevalence (100%) was earlier reported in India (Murugadas et al., 2016), and prevalence of 90% and 100% was reported in Egypt (Elkamouny et al., 2020; Anjusha et al., 2022, respectively), whereas the lowest prevalence (14.43% and 60%) was reported by prior studies on *S. aureus* isolated from fish (Mohammed et al., 2020; Aksakal et al., 2022, respectively). With regard to our finding, the prevalence of *mec* gene (60%) was higher than those in prior studies that reported prevalence of 44% (Elkamouny et al., 2020), 10.30% (Mohammed et al., 2020), 47.5% (Aksakal et al., 2022), and 1.8% (Anjusha et al., 2022) in *S. aureus* isolated from fish but lower than the 100% reported in India (Murugadas et al., 2016).

Identifying risk factors related to the proportion of positive bacterial isolates would be beneficial for preventing cross-contamination and improving fish quality. Men had a higher prevalence of *Aeromonas* spp., *A. hydrophila*, and MRSA than women, with no significant differences. These findings corresponded with those of Grave et al. (2022), who found that men were more likely to be infected with *Aeromonas* (56%) than women (44%). MRSA colonization rates in men are greater than in women, according to Liu et al. (2015). The main explanations were handwashing and hand hygiene practices, which had greater proportions in women than men (76% vs 57% and 59% vs 32%, respectively) (Mackert et al., 2013) and might be responsible since estrogen has a protective impact on women and immunological responses seem to be involved in the variations between the sexes (Humphreys et al., 2015). The age category of >30 years showed the highest prevalence rate of *Aeromonas* spp., *A. hydrophila*, *S. aureus*, and MRSA, compared with the other two age categories. Our findings are consistent with those of Hiroyuki et al. (2001), who found that older age groups had the highest prevalence rate of MRSA. According to Yuwono et al. (2021), a prior study found a strong (p < 0.001) correlation between the isolation rate of *Aeromonas* and increasing age. Immune system failure, malnutrition, and physiological changes are associated with an increased susceptibility to infections in older age groups compared to those in younger age groups (Yoshikawa, 2000). In relation to their job title and daily work hours, fish processors were found to have higher positivity rates for *Aeromonas* species, *S. aureus*, and MRSA than fish sellers. This may be because fish processors do not wear gloves during work and engage in a variety of unhygienic handling practices, such as washing fish in contaminated water or processing multiple fish with a single knife and chopping board and rarely cleaning or disinfecting in between (Bedane et al., 2022). In terms of handwashing and other hygiene practices, the non-washing hands group had a greater percentage of all isolated bacteria than the washing hands group. As a result, compared to the group that washed their hands three times a day (0%), the group that washed their hands once a day had the greatest reported bacterial prevalence. Lower bacterial positivity was found in the hand sanitizer–using group compared to that in the nonsanitizer-using group due to hand hygiene practice, our finding were corroborated by Burton et al. (2011), who concluded that handwashing with water and non-antibacterial soap is more effective than handwashing with water alone. Human skin contamination with *S. aureus* was significantly reduced when hand sanitizer was used (Bondurant et al., 2020). Enhancing hand hygiene is necessary to stop the spread of pathogens and has been acknowledged as a crucial public health intervention (Ejemot et al., 2008). The group with a history of skin infections had a higher percentage of all isolated bacteria than the group without a history of skin infections. Furthermore, there was no statistically significant difference in the prevalence of any of the four tested bacteria between the groups labeled as “using unprescribed antibiotics” and “not using antibiotics.” The cause could be a severe health issue called “infection recurrence,” which is defined as the emergence of infection-related symptoms after more than 7 days following a negative blood culture and a clinically noticeable improvement. Recurrent episodes are brought on by the same strain of the original infection (Liao et al., 2010). When sanitizer was used to clean instruments, the prevalence rates of *Aeromonas* spp., *A. hydrophila*, *S. aureus*, and MRSA were lower in the group that used sanitizer than in the group that did not. Chopping boards, containers, and knives are regularly used for extended periods of time at fish markets without being cleaned or disinfected. These contaminated surfaces are referred to as fomites, and pathogens can endure months of survival there. The bacteria that were present the day before might have multiplied if cleaning supplies had not been available. As a result, fomites act as a second reservoir and spread infections among hosts; however, following cleaning, those contact surfaces may be rated as good and acceptable (Martowitono, 2011; Artasensi et al., 2021).

An investigation was conducted into the antibiotic resistance pattern of *A. hydrophila* against 10 antimicrobial drugs (eight classes). According to previous research, all *A. hydrophila* isolates demonstrated 100% AXE resistance from fish in Ethiopia (Kerigano et al., 2023), Egypt (Elkamouny et al., 2020), and Sri Lanka (Dhanapala et al., 2021). Furthermore, our findings indicated that *A. hydrophila* was more resistant to TE, SXT, and ENR. This is consistent with assessments of resistance to ENR (73.3%), ERY (53.3%), and SXT (80%) made by Dhanapala et al. (2021). Fish resistances to TE and SXT were higher in Kafr-Elsheikh (Sherif and Kassab, 2023) and Mansoura City (Elkamouny et al., 2020) than in Damietta Governorate (Ahmed et al., 2018). Fish resistance to AXE, TE, and NA was also higher (76% each). Chloramphenicol and gentamicin were the most effective antibiotics against *A. hydrophila*, which is consistent with the findings of Dhanapala et al. (2021) and Kerigano et al. (2023), who reported a 100% susceptibility to C. However, Martins et al. (2023) found that although *A. hydrophila* were significantly resistant to AM (>89%), they were highly susceptible to TE (89.5%), CIP (78.9%), C and NA (68.4%), and AXE (47.4%). In addition, *A. hydrophila* has better resistance to C (60%) (Hafez et al., 2018) but was sensitive to SXT (56%) (Ahmed et al., 2018). CIP and GN had an effect on *A. hydrophila*, but these strains were not susceptible to AM (Ramadan et al., 2018; Hassan et al., 2020). The production of the lactamase enzyme through the expression of chromosomal lactamases may be the cause of *A. hydrophila*’s highest resistance to amoxicillin (Hafez et al., 2018).

The isolates of *S. aureus* exhibited a resistance profile that was most resistant to AM, followed by NA and AXE, but was highly sensitive to GN and TE. These results are strikingly similar to those of Elkamouny et al. (2020), who discovered that *S. aureus* was highly susceptible to GN (86%), TE (72%), ENR (50%), and C (44%) but extremely resistant to CIP and SXT. Contrary to our findings, Lim et al. (2023) reported resistance of S. aureus to TE (83 %); GN (25 %) and CIP (1.7 %) but agreed with our finding in their resistance to AM and sensitivity to C in Singapore. Furthermore, *S. aureus* has strong resistance to SXT (82%), AM (53%), and ERY (49%) in Northern Greece, which is in line with our findings. However, the findings of Abrahima et al. (2010) disagreed with our findings about *S. aureus*’s resistance to GN and C (65% each).

All MRSAs under investigation showed high rates of resistance to AXE and AM, as well as high levels of sensitivity to C (100%). Our results confirmed those of earlier research conducted in Nigerian fish markets by Grema et al. (2015), who found that MRSA exhibited high resistance to CIP (94.7%), GN (89.5%), AXE (76.3%), and TE (68.4%). Furthermore, in Indian seafood markets, Sivaraman et al. (2022) discovered that MRSA had the highest resistance to AM and GN, while resistance to SXT was the highest. Furthermore, Fri et al. (2020) reported that on healthy edible fish, the maximum susceptibility to C. (100%), GN and CIP (94% each), and AM (67%) was noted.

Bacteria that exhibit MDR make it more difficult to manage infections in humans and animals (Ibrahim et al., 2022b; Elmowalid et al., 2022). Unfortunately, our findings demonstrate that a significant portion of the isolates of bacteria have MDR. Nonetheless, the MDR of *A. hydrophila* was lower than 88.37% (Odeyemi and Ahmad, 2017) but greater than the 60% MDR prevalence observed in freshwater fish (Fauzi et al., 2021). A higher prevalence of MDR *S. aureus* was found than in earlier studies, which found 66% in Egypt (Elkamouny et al., 2020), 90.6% in China (Rong et al., 2017), and 71.4% in Mumbo et al. (2023). Additionally, all isolates had MAR indexes greater than 0.2, suggesting that high-risk sources of contamination, growing resistance among the bacterial strains under investigation, and antimicrobial misuse were present. Alarmingly high rates of multidrug resistance were found in the isolates collected for this investigation. Our findings are consistent with those of Usui et al. (2016), who reported that fish constitute a dangerous source of contamination, with MAR index values ranging from 0.11 to 0.88 and an average of 0.489 or higher (De Silva et al., 2019).

Multidrug-resistant bacteria are a serious problem from a hygiene standpoint, which affect public health and the global economy through higher rates of infection-related morbidity and mortality, longer illness durations, and high costs (Peña et al., 2011). According to Duedu et al. (2017), eating raw or undercooked fish can expose consumers to these bacteria and lead to colonization and infection. Furthermore, the resistance characteristic may be passed to different microbes and dispersed throughout environments by means of insects, other vectors, and vehicles (Donkor, 2019; Obeng-Nkrumah et al., 2019).

## 5 Conclusion

In conclusion, most risk factors were significantly correlated with the prevalence of *A. hydrophila*, *S. aureus*, and MRSA recovered from retail fish markets in Egypt. Our findings demonstrated that fish markets lack proper handling procedures and hygienic standards for food safety. Furthermore, among fish bacterial isolates, MDR patterns represented a high-risk concern. These results offered awareness information concerning handlers’ and consumers’ health in addition to marketed fish quality. Collaboration between veterinary authorities and public health professionals is necessary to control the spread and transmission of these bacterial pathogens. Our findings recommend regular training for fish handlers regarding safe applied food handling procedures.

## Data availability statement

The original contributions presented in the study are included in the article/**Supplementary Material**. Further inquiries can be directed to the corresponding authors.

## Ethics statement

The study was reviewed and approved by the Institutional Use Committee (IACUC) of Zagazig University (Ref. No.: ZU-IACUC/2/F/233/2023). The studies were conducted in accordance with the local legislation and institutional requirements. The participants provided their written informed consent to participate in this study. The study was reviewed and approved by the Institutional Animal Care and Use Committee (IACUC) of Zagazig University (Ref. No.: ZU-IACUC/2/F/233/2023). The study was conducted in accordance with the local legislation and institutional requirements.

## Author contributions

ASA: Conceptualization, Data curation, Formal analysis, Investigation, Methodology, Project administration, Resources, Software, Supervision, Validation, Writing – original draft, Writing – review & editing. RA: Conceptualization, Investigation, Methodology, Resources, Writing – review & editing. NE: Conceptualization, Investigation, Methodology, Resources, Writing – review & editing. SE: Conceptualization, Data curation, Investigation, Resources, Supervision, Writing – review & editing. AA: Conceptualization, Data curation, Funding acquisition, Investigation, Writing – review & editing. HA: Conceptualization, Funding acquisition, Investigation, Project administration, Validation, Writing – review & editing. DA: Conceptualization, Data curation, Funding acquisition, Investigation, Writing – review & editing. WH: Data curation, Formal analysis, Visualization, Writing – review & editing. HD: Formal analysis, Investigation, Methodology, Writing – original draft, Writing – review & editing. DI: Conceptualization, Data curation, Formal analysis, Visualization, Methodology, Writing – original draft, Writing – review & editing.
