# Supplementary material for: Cross-sectional analysis of risk factors associated with Mugil cephalus in retail fish markets concerning methicillin-resistant Staphylococcus aureus and Aeromonas hydrophila
Source: Front Cell Infect Microbiol. 2024 Feb 2;14:1348973. doi: 10.3389/fcimb.2024.1348973 (PMC10869461; doi:10.3389/fcimb.2024.1348973)
Supplement: Supplementary file 1 [file DataSheet_1.docx]

Supplementary Material

# Supplementary Table 1 Frequency of antimicrobial resistance of *A. hydrophila, S.aureus and Staph. MRSA* in different sources in examined fish markets.

| Source | Antibiotic resistance patterns (%) | | | | | | | | | | | | | | | | | |  | |
| --- | --- | --- | --- | --- | --- | --- | --- | --- | --- | --- | --- | --- | --- | --- | --- | --- | --- | --- | --- | --- |
|  | **ERY** | | **NA** | | **CIP** | | **C** | | **TE** | | **SXT** | | **AXE** | | **AM** | | **EN** | | **GN** | |
| *Aeromonas hydrophila* | | | | | | | | | | | | | | | | | | | | |
| Fish skin (20) | 10(50) | | 13(65) | | 2(10) | | 0(0) | | 18(90) | | 17(85) | | 20(100) | | 3(15) | | 15(75) | | 2(10) | |
| Fish muscle (5) | 2(40) | | 3(60) | | 1(20) | | 0(0) | | 5(100) | | 4(80) | | 5(100) | | 2(40) | | 4(80) | | 1(20) | |
| Washing water (7) | 4(57.1) | | 5(71.43) | | 1(14.3) | | 0(0) | | 5(71.43) | | 4(57.1) | | 7(100) | | 1(14.3) | | 5(71.4) | | 0(0) | |
| Chopping board (3) | 2(66.67) | | 0(0) | | 0(0) | | 0(0) | | 2(66.67) | | 2(66.67) | | 3(100) | | 1(33.3) | | 1(33.3) | | 0(0) | |
| Knifes (4) | 2(50) | | 3(75) | | 1(25) | | 0(0) | | 4(100) | | 4(100) | | 4(100) | | 1(100) | | 4(100) | | 1(25) | |
| Hands (4) | 2(50) | | 2(50) | | 1(25) | | 0(0) | | 4(100) | | 3(75) | | 4(100) | | 1 (25) | | 4(100) | | 1(25) | |
| Total (43) | **22(51.16)** | | **26(60.46)** | | **6(13.95)** | | **0(0)** | | **38(88.37)** | | **34(79.07)** | | **43(100)** | | **9(20.9)** | | **33(76.74)** | | **5(11.63)** | |
| *S.aureus* | | | | | | | | | | | | | | | | | | | | |
| Fish skin (34) | | 7(20.58) | | 30(88.24) | | 23(67.65) | | 7(20.58) | | 4(11.76) | | 20(58.82) | | 26(76.47) | | 34(100) | | 9(26.47) | | 4(11.76) |
| Fish muscle (6) | | 2(33.3) | | 4(66.67) | | 6(100) | | 2(33.3) | | 2(33.3) | | 5(83.33) | | 4(66.67) | | 6(100) | | 3(50) | | 1(16.67) |
| Washing water (12) | | 4(33.3) | | 11(91.67) | | 7(58.33) | | 2(16.66) | | 1(8.33) | | 7(58.33) | | 6(50) | | 12(100) | | 3(25) | | 2(16.67) |
| Chopping board (8) | | 4(50) | | 6(75) | | 6(75) | | 0(0) | | 1(12.5) | | 5(62.5) | | 6(75) | | 8(100) | | 4(50) | | 1(12.5) |
| Knifes (12) | | 4(33.33) | | 9(75) | | 5(41.67) | | 0(0) | | 1(8.33) | | 7(58.33) | | 9(75) | | 12(100) | | 4(33.3) | | 2(16.67) |
| Hands (12) | | 7(58.33) | | 8(66.67) | | 8(66.67) | | 3(25) | | 4(33.3) | | 8(66.67) | | 9(75) | | 12(100) | | 5(41.67) | | 3(25) |
| Total (84) | | **28(33.33)** | | **68(80.95)** | | **55(65.48)** | | **14(16.67)** | | **13(15.48)** | | **52(61.9)** | | **60(71.42)** | | **84(100)** | | **28(33.3)** | | **13(15.48)** |
| *Staph.MRSA* | | | | | | | | | | | | | | | | | | | | |
| Fish skin (4) | 2(50) | | 4(100) | | 4(100) | | 0(0) | | 2(50) | | 0(0) | | 4(100) | | 4(100) | | 4(100) | | 3(75) | |
| Fish muscle (1) | 0(0) | | 0(0) | | 0(0) | | 0(0) | | 1(100) | | 0(0) | | 1(100) | | 1(100) | | 0(0) | | 1(100) | |
| Washing water (3) | 0(0) | | 2(66.6) | | 2(66.6) | | 0(0) | | 2(66.6) | | 0(0) | | 3(100) | | 3(100) | | 2(66.6) | | 3(100) | |
| Chopping board (2) | 0(0) | | 2(100) | | 2(100) | | 0(0) | | 0(0) | | 1(50) | | 2(100) | | 2(100) | | 2(100) | | 1(50) | |
| Knifes (2) | 0(0) | | 2(100) | | 2(100) | | 0(0) | | 1(50) | | 0(0) | | 2(100) | | 2(100) | | 2(100) | | 2(100) | |
| Hands (3) | 1(33.3) | | 3(100) | | 3(100) | | 0(0) | | 2(66.6) | | 1(33.3) | | 3(100) | | 3(100) | | 3(100) | | 3(100) | |
| Total (15) | **3(20)** | | **13(86.66)*** | | **13(86.66)*** | | **0(0)** | | **8(53.33)** | | **1(6.66)** | | **15(100)** | | **15(100)** | | **13(86.66)*** | | **13(86.66)** | |

* Significant level of Chi-Square value was considered at P ˂ 0.05.

ampicillin )AM(; chloramphenicol )C(; ciprofloxacin )CIP(; enrofloxacin )ENR(; erythromycin )ERY(; nalidixic acid )NA(; gentamicin )GN(; amoxicilline )AXE(; tetracycline )TE(;trimethoprim/sulfamethoxazole )SXT(

| **8** | **7** | **6** | **5** | **4** | **3** | **2** | **1** | **P** | **N** | **L** |
| --- | --- | --- | --- | --- | --- | --- | --- | --- | --- | --- |
|  | | | | | | | | | | |


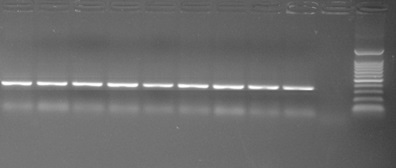


**326 bp**

**A**


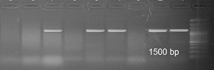


**B**

**Supplementary Figure 1** Exampler for agarose gel electrophoresis images representing amplification of *A.hydrophila* genes recovered from different sources in retailed fish markets (A) aerolysin (*aerA*) gene (326 bp), (B) hemolysins (*hlyA*)gene (1500 bp), using simplex PCR.. Lane L: DNA ladder (100 bp), Lanes P and N: represent positive and negative controls, respectively. Lane (1-8): represent positive and negative examined samples.

| **8** | **7** | **6** | **5** | **4** | **3** | **2** | **1** | **P** | **L** |
| --- | --- | --- | --- | --- | --- | --- | --- | --- | --- |
|  | | | | | | | | | |


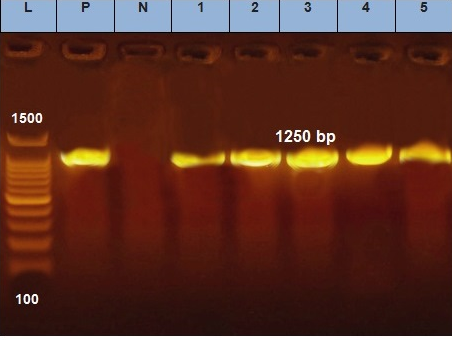

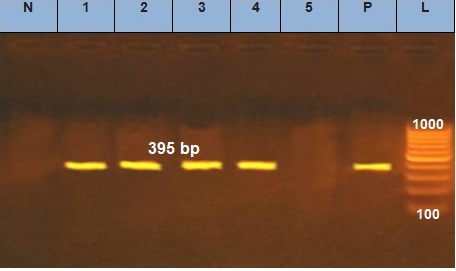

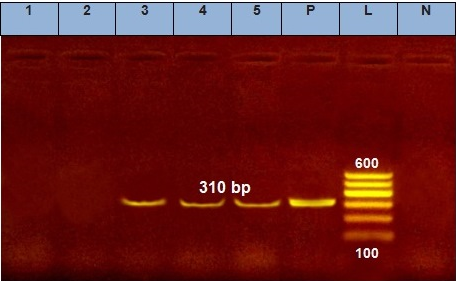


**A**

**B**

**C**

**Supplementary Figure 2** Exampler for agarose gel electrophoresis images representing amplification of *S.aureus* genes recovered from different sources in retailed fish markets (A) genus-specific (23S rRNA), (B) species-specific (*nuc*) and (C) methicillin resistance (*mecA*) in *S. aureus* isolates using simplex PCR.. Lane L: DNA ladder (100 bp), Lanes P and N: represent positive and negative controls, respectively. Lane (1-5): represent positive and negative examined samples
